# The Story of the Insane from Year to Year

**Published:** 1899-08-26

**Authors:** 


### THE STORY OF THE INSANE FROM YEAR TO YEAR.

(Eleventh Series.)

### THE BRISTOL CITY ASYLUM.

In this asylum the numbers rose during 1897 from 734 to 751. There were 191 first admissions, 33 readmissions, 60 recoveries, 75 discharged relieved, and 72 deaths. The recoveries stand at 26·78 per cent. on the admissions, which is considerably lower than their own average and that of public asylums generally; but the percentage is at 37·34 in another part of the report. The deaths stand at 9·84 on the average number resident. Only 12 of the deaths were caused by cerebral diseases, but consumption was the cause in 13 cases, pneumonia in 6, and enteritis in 2. Moral causes account for the insanity in 8 of the year's admissions, hereditary tendency in 48, and drink in 18, while in 40 there was no known cause. As already stated, there were 72 deaths, and we are informed that post-mortem examinations were made in 67 instances. Dr. Benham has some very sound remarks on the way asylum authorities are blamed, sometimes for detaining patients too long, and sometimes for discharging them too soon, and he thinks the re-certification clause in the last Lunacy Act may be one reason why the latter occasionally happens. The clause was beyond doubt a most useless, senseless encumbrance to our lunacy statutes, and it is not at all unlikely that it may have been responsible for dangerous lunatics being thrown on the public. The asylum is being added to, and when the extensions are finished there will be room for 941 patients, which ought to be fixed as the limit beyond which no further extension is desirable. The visitors "record their satisfaction with the manner in which the work of the asylum has been carried on by the medical superintendent."

### JOINT COUNTIES ASYLUM, CARMARTHEN.

On January 1st, 1897, this asylum contained 606 patients, and the numbers had risen to 625 on the last day of December There were 115 first admissions, 5 readmissions, 32 recoveries, 10 discharged relieved, and 55 deaths. These numbers show that the recoveries were 34·7 per cent. of the admissions, and that the deaths were 8·87 per cent. of the average number resident. Cerebral and spinal diseases account for 9 of the deaths, and consumption for 14. It would be desirable if Table V. were arranged in a similar way as in other asylums. Of the year's admissions 15 were caused by various moral causes, including domestic trouble, religion, and mental worry; 19 by intemperance in drink, and 33 by hereditary tendency. It is proposed to build a detached hospital for isolating infectious diseases, but provision is only to be made for three men and three women. Judging from what has happened in other asylums this number of beds would be nearly useless, unless, indeed, the kitchen and laundry be made of large size, and materials kept always on hand for the erection of wooden huts, tents, or iron buildings. Dr. Goodall does not think the mortality from consumption excessive, and he favours the idea that consumption is a mode of exitus in the congenitally deficient, and that it is less a matter of environment than of inherited tendency. We, of course, agree so far with Dr. Goodall as to admit that tubercular disease is one mode of exit in the insane; but we think he should substitute the word "more" for the word "less" in the last part of the sentence. The instances he refers to of the model sanitation asylums having a mortality from consumption of 44 per cent., and of the overcrowded asylum having a consumptive mortality of only 6 per cent., do not of themselves prove anything. In the first case the patients may not get their 3,000 cubic feet of air per hour, and they may spend only a small part of the day in the open air. In the second case the perflation of air in the dormitories may give more than the minimum 3,000 cubic feet per patient, and thus patients may be out of doors nearly all day. In the first case there would be real overcrowding, in the second case only nominal overcrowding. Again, the first asylum may be warmed by some injurious system of hot air or hot water, the second may be warmed by health-giving open fireplaces. Having been informed on these points we should want to know about the diet, the clothing, the medical attendance, &c. Dr. Goodall takes a somewhat hopeful view of the establishment of pathological laboratories, and we trust to find in some future report that his views will be justified. As regards prevention of insanity he says "that can be preached by the lunacy specialist with at least as much eloquerce as by the general physician, and probably with as little efficacy." From which we may infer that his hopes of prevention are not great. The committee "bear testimony to the most able and satisfactory work which is being continued by the medical superintendent."

